# Supplementary material for: Explainable mortality prediction models incorporating social health determinants and physical frailty for heart failure patients
Source: PLoS One. 2025 Sep 3;20(9):e0327979. doi: 10.1371/journal.pone.0327979 (PMC12407480; doi:10.1371/journal.pone.0327979)
Supplement: S1 File — (DOCX) [file pone.0327979.s001.docx]

**Explainable Mortality Prediction Models Incorporating Social Health Determinants and Physical Frailty for Heart Failure Patients**

Additional Supporting Information can be found:

S1 Table. Feature names

S2 Table. Missing ratio of the in-hospital cohort and discharge hospital cohort

S3 Table. Cohort baseline (in-hospital: survivor vs. non-survivor)

S4 Table. Cohort baseline (discharge hospital: survivor vs. non-survivor within 90 days)

S5 Table Cohort baseline (discharge hospital: survivor vs. non-survivor within 1 year)

S6 Table. Detailed model performance for short-term outcomes of internal test set and external test set (95% CI)

S7 Table. Features ranking of short- and long-term outcome prediction

S8 Table. Comparison of the models’ performance incorporating with SDOH, physical frailty and lung sounds

S9 Table. The performance changing of including part of features (AUROC)

S10 Table. Comparison of the models’ performance incorporating with data measured at the first and last day in ICU

S1 Fig. The interpretation of in-hospital, 90-day, and 1-year mortality risk prediction models (non-survivor and survivor cases)

**S1 Table. Feature names**

| Type (number) | Feature name (In hospital)  (n=79) | Feature name (out of hospital)  (n=122) |
| --- | --- | --- |
| Basic information (8/10) | Age, Gender, Weight, BMI, CCI score, Days before ICU admission, Admission type, Elective surgery | Age, Gender, Weight, BMI, CCI score, Days before ICU admission, Admission type, Elective surgery, weight(leave), weight difference |
| Social determinants of health (4/4) | Primary language, Martial status, Insurance, Ethnicity | Primary language, Martial status, Insurance, Ethnicity |
| Vital signs (8/15) | SBP, MBP, Respiration rate, Temperature, Heart rate, GCS, SpO2, FiO2 | SBP, MBP, Respiration rate, Temperature, Heart rate, GCS, SpO2, FiO2, SBP(leave),, MBP(leave), Respiration rate(leave), Temperature(leave), Heart rate(leave), SpO2(leave), GCS(leave) |
| Laboratory tests (31/48) | Creatinine, Bilirubin, Platelet, BUN, White blood cell, Glucose, Hematocrit, Potassium, Sodium, Bicarbonate(max), Bicarbonate(min), Albumin, ALT, AST, ALP, PT, PTT, INR, Hemoglobin, Chloride, Lactate, Base Excess, Troponin, Lymphocytes(max), Lymphocytes(min), Neutrophils, Magnesium, Anion gap, PaO2, PaCO2, SpO2/FiO2 | Creatinine, Bilirubin, Platelet, BUN, White blood cell, Glucose, Hematocrit, Potassium, Sodium, Bicarbonate(max), Bicarbonate(min), Albumin, ALT, AST, ALP, PT, PTT, INR, Hemoglobin, Chloride, Lactate, Base Excess, Troponin, Lymphocytes(max), Lymphocytes(min), Neutrophils, Magnesium, Anion gap, PaO2, PaCO2, SpO2/FiO2, PaO2/FiO2, Creatinine(leave), Platelet(leave), BUN(leave), White blood cell(leave), Glucose(leave), Hematocrit(leave), Potassium(leave), Sodium(leave), Bicarbonate(max)(leave), Bicarbonate(min)(leave), PT(leave), PTT(leave), INR(leave), Hemoglobin(leave), Chloride(leave), Magnesium(leave), Anion gap(leave) |
| Outputs (1/2) | Urine outputs | Urine outputs, Urine outputs(leave) |
| Treatments (4/8) | Mechanical ventilation, Vasopressor, Milrinone, Furosemide | Mechanical ventilation, Vasopressor, Milrinone, Furosemide, Mechanical ventilation(leave), Vasopressor(leave), Milrinone(leave), Furosemide(leave) |
| Physical frailty (5/10) | Activity, Fall risk, Braden nutrition, Braden activity, Braden mobility | Activity, Fall risk, Braden nutrition, Braden activity, Braden mobility, Activity(leave), Fall risk(leave), Braden nutrition(leave), Braden activity(leave), Braden mobility(leave) |
| Lung sounds (4/8) | LLL lung sounds, LUL lung sounds, RLL lung sounds, RUL lung sounds | LLL lung sounds, LUL lung sounds, RLL lung sounds, RUL lung sounds, LLL lung sounds(leave), LUL lung sounds(leave), RLL lung sounds(leave), RUL lung sounds(leave) |
| Missing value indicator (14/17) | Bilirubin(flag), Albumin(flag), ALT(flag), AST(flag), ALP(flag), Lactate(flag), Base excess(flag), FiO2(flag), PaO2(flag), PaCO2(flag) Troponin(flag), Lymphocytes(max)(flag),  Lymphocytes(min)(flag), Neutrophils(flag) | Bilirubin(flag), Albumin(flag), ALT(flag), AST(flag), ALP(flag), Lactate(flag), Base excess(flag), FiO2(flag), PaO2(flag), PaCO2(flag) Troponin(flag), Lymphocytes(max)(flag),  Lymphocytes(min)(flag), Neutrophils(flag), PT(leave)(flag), PTT(leave)(flag), INR(leave)(flag) |
| ***Abbreviations****: BMI, body mass index; CCI, Charlson comorbidity index; SBP, systolic blood pressure; MBP, mean blood pressure; GCS, Glasgow coma score; SpO2, oxygen saturation; FiO2, fraction of inspired oxygen; PaO2,* *partial pressure of oxygen; PaCO2, pressure of carbon dioxide; SpO2/FiO2, oxygenation index; BUN, blood urea nitrogen; ALT, alanine aminotransferase; AST, aspartate transaminase; ALP, alkaline phosphatase; PT, prothrombin time; PTT, partial thromboplastin time; INR, international normalized ratio; RUL, right upper lobe; LUL, left upper lobe; RLL, right* *lower lobe; LLL, left lower lobe; (flag), the missing value indicator.* | | |

**S2 Table. Missing ratio of the in-hospital cohort and discharge hospital cohort**

|  | **In-hospital** | | **Discharge hospital** | |
| --- | --- | --- | --- | --- |
|  | **Train set (%)** | **Test set (%)** | **Train set (%)** | **Test set (%)** |
| **Demographics** | | | | |
| Age | 0 | 0 | 0 | 0 |
| Gender | 0 | 0 | 0 | 0 |
| Weight | 3.39 | 3.46 | 3.39 | 3.46 |
| BMI | 33.78 | 35.39 | 33.78 | 35.39 |
| Elective surgery | 0 | 0 | 0 | 0 |
| CCI score | 0 | 0 | 0 | 0 |
| Length of stay before ICU | 0 | 0 | 0 | 0 |
| Admission type | 0 | 0 | 0 | 0 |
| Weight (leave) |  |  | 1.85 | 1.75 |
| Weight difference |  |  | 1.85 | 1.75 |
| **Social determinants of health** | | | | |
| Primary language | 0 | 0 | 0 | 0 |
| Insurance | 0 | 0 | 0 | 0 |
| Ethnicity | 0 | 0 | 0 | 0 |
| Marital status | 0 | 0 | 0 | 0 |
| **Treatments** | | | | |
| Mechanical ventilation | 0 | 0 | 0 | 0 |
| Vasopressor | 0 | 0 | 0 | 0 |
| Milrinone | 0 | 0 | 0 | 0 |
| Furosemide | 0 | 0 | 0 | 0 |
| Mechanical ventilation (leave) |  |  | 0 | 0 |
| Vasopressor (leave) |  |  | 0 | 0 |
| Milrinone (leave) |  |  | 0 | 0 |
| Furosemide (leave) |  |  | 0 | 0 |
| **Laboratory tests** | | | | |
| Creatinine (max) | 0.13 | 0.16 | 0.13 | 0.16 |
| Bilirubin (max) | 49.39 | 49.2 | 49.39 | 49.2 |
| Platelet (min) | 0.41 | 0.27 | 0.41 | 0.27 |
| BUN (max) | 0.16 | 0.23 | 0.16 | 0.23 |
| White blood cell (max) | 0.48 | 0.31 | 0.48 | 0.31 |
| Glucose (max) | 0.29 | 0.43 | 0.29 | 0.43 |
| Hematocrit (max) | 0.19 | 0.19 | 0.19 | 0.19 |
| Potassium (max) | 0.16 | 0.27 | 0.16 | 0.27 |
| Sodium (max) | 0.16 | 0.23 | 0.16 | 0.23 |
| Bicarbonate (min) | 0.38 | 0.27 | 0.38 | 0.27 |
| Bicarbonate (max) | 0.38 | 0.27 | 0.38 | 0.27 |
| SpO2/FiO2 | 0 | 0 | 0 | 0 |
| Albumin (min) | 60.11 | 60.64 | 60.11 | 60.64 |
| ALT (max) | 48.98 | 48.62 | 48.98 | 48.62 |
| AST (max) | 48.88 | 48.54 | 48.88 | 48.54 |
| ALP (max) | 49.78 | 49.36 | 49.78 | 49.36 |
| PT (max) | 6.94 | 8.32 | 6.94 | 8.32 |
| PTT (max) | 7.25 | 8.21 | 7.25 | 8.21 |
| INR (min) | 6.92 | 8.32 | 6.92 | 8.32 |
| Hemoglobin (min) | 0.3 | 0.27 | 0.3 | 0.27 |
| Chloride (min) | 0.31 | 0.23 | 0.31 | 0.23 |
| PaO2 (min) | 34.87 | 37.15 | 34.87 | 37.15 |
| PaCO2 (max) | 34.87 | 37.15 | 34.87 | 37.15 |
| Lactate (max) | 52.94 | 54.53 | 52.94 | 54.53 |
| Base excess (min) | 34.87 | 37.15 | 34.87 | 37.15 |
| Troponin (max) | 56.91 | 55.08 | 56.91 | 55.08 |
| Lymphocytes (max) | 54.2 | 55.43 | 54.2 | 55.43 |
| Lymphocytes (min) | 54.2 | 55.43 | 54.2 | 55.43 |
| Neutrophils (min) | 53.93 | 55.31 | 53.93 | 55.31 |
| Magnesium (max) | 3.28 | 3.31 | 3.28 | 3.31 |
| Anion gap (max) | 1.51 | 1.56 | 1.51 | 1.56 |
| Creatinine (max)(leave) |  |  | 5.31 | 5.41 |
| Platelet (min)(leave) |  |  | 6.41 | 5.95 |
| BUN (max)(leave) |  |  | 5.34 | 5.52 |
| White blood cell (max)(leave) |  |  | 6.49 | 5.95 |
| Glucose (max)(leave) |  |  | 6.63 | 6.85 |
| Hematocrit (max)(leave) |  |  | 5.79 | 5.52 |
| Potassium (max)(leave) |  |  | 5.79 | 5.87 |
| Sodium (max)(leave) |  |  | 5.74 | 5.41 |
| Bicarbonate (min)(leave) |  |  | 5.83 | 5.6 |
| Bicarbonate (max)(leave) |  |  | 5.83 | 5.6 |
| PT (max)(leave) |  |  | 35.38 | 36.6 |
| PTT (max)(leave) |  |  | 36.38 | 35.59 |
| INR (min)(leave) |  |  | 35.4 | 36.64 |
| Hemoglobin (min)(leave) |  |  | 6.6 | 5.99 |
| Chloride (min)(leave) |  |  | 5.74 | 5.41 |
| Magnesium (max)(leave) |  |  | 8.66 | 8.87 |
| Anion gap (max)(leave) |  |  | 6.67 | 6.5 |
| **Urine output** | | | | |
| Urine output | 2.61 | 1.94 | 2.61 | 1.94 |
| Urine output (leave) |  |  | 5.67 | 5.1 |
| **Vital signs** | | | | |
| GCS (min) | 0 | 0 | 0 | 0 |
| Heart rate (mean) | 0 | 0 | 0 | 0 |
| MBP (mean) | 0 | 0 | 0 | 0 |
| SBP (mean) | 0 | 0 | 0 | 0 |
| Respiratory rate (mean) | 0 | 0 | 0 | 0 |
| Temperature (mean) | 0 | 0 | 0 | 0 |
| SpO2 (min) | 0 | 0 | 0 | 0 |
| FiO2 (max) | 52.52 | 54.84 | 52.52 | 54.84 |
| GCS (min)(leave) |  |  | 0.53 | 0.47 |
| Heart rate (mean)(leave) |  |  | 0.23 | 0.12 |
| MBP (mean)(leave) |  |  | 0.43 | 0.35 |
| SBP (mean)(leave) |  |  | 0.56 | 0.47 |
| Respiratory rate (mean)(leave) |  |  | 0.43 | 0.35 |
| Temperature (mean)(leave) |  |  | 0.92 | 0.82 |
| SpO2 (min)(leave) |  |  | 0.64 | 0.43 |
| **Physical frailty** | | | | |
| Activity | 0.49 | 0.35 | 0.49 | 0.35 |
| Fall risk | 0 | 0 | 0 | 0 |
| Braden nutrition | 1.03 | 1.28 | 1.03 | 1.28 |
| Braden mobility | 1.01 | 1.28 | 1.01 | 1.28 |
| Braden activity | 1.02 | 1.32 | 1.02 | 1.32 |
| Activity (leave) |  |  | 1.06 | 0.82 |
| Fall risk (leave) |  |  | 0 | 0 |
| Braden nutrition (leave) |  |  | 4.72 | 4.32 |
| Braden mobility (leave) |  |  | 4.71 | 4.32 |
| Braden activity (leave) |  |  | 4.71 | 4.32 |
| **Lung sounds** | | | | |
| LLL lung sounds | 0.11 | 0.12 | 0.11 | 0.12 |
| LUL lung sounds | 0.1 | 0.12 | 0.1 | 0.12 |
| RLL lung sounds | 0.1 | 0.12 | 0.1 | 0.12 |
| RUL lung sounds | 0.09 | 0.08 | 0.09 | 0.08 |
| LLL lung sounds (leave) |  |  | 1.07 | 0.82 |
| LUL lung sounds (leave) |  |  | 1.03 | 0.78 |
| RLL lung sounds (leave) |  |  | 1.05 | 0.86 |
| RUL lung sounds (leave) |  |  | 1.01 | 0.78 |

*Note: variable (leave) represents that the data was collected at the last day in ICU.*

**S3 Table. Cohort baseline (in-hospital: survivor vs. non-survivor)**

|  | **Overall**  **(n=12,586)** | **Survivor**  **(n=10,990)** | **Non-survivor**  **(n=1,866)** | **P-Value** |
| --- | --- | --- | --- | --- |
| **Age, median [Q1,Q3]** | 75.0 [65.0,84.0] | 74.0 [64.0,83.0] | 79.0 [70.0,86.0] | <0.001 |
| **Female, n (%)** | 5957 (46.3) | 5054 (46.0) | 903 (48.4) | 0.057 |
| **Admission type, n (%)** |  |  |  | <0.001 |
| **ELECTIVE** | 964 (7.5) | 913 (8.3) | 51 (2.7) |  |
| **EMERGENCY** | 11892 (92.5) | 10077 (91.7) | 1815 (97.3) |  |
| **Ethnicity, n (%)** |  |  |  | <0.001 |
| **Asian** | 257 (2.0) | 220 (2.0) | 37 (2.0) |  |
| **Black** | 1141 (8.9) | 1021 (9.3) | 120 (6.4) |  |
| **Hispanic** | 303 (2.4) | 267 (2.4) | 36 (1.9) |  |
| **Other** | 2009 (15.6) | 1622 (14.8) | 387 (20.7) |  |
| **White** | 9146 (71.1) | 7860 (71.5) | 1286 (68.9) |  |
| **Marital status, n (%)** |  |  |  | 0.006 |
| **Divorced** | 915 (7.1) | 805 (7.3) | 110 (5.9) |  |
| **Married** | 5905 (45.9) | 5083 (46.3) | 822 (44.1) |  |
| **Unknown** | 3150 (24.5) | 2681 (24.4) | 469 (25.1) |  |
| **Widowed** | 2886 (22.4) | 2421 (22.0) | 465 (24.9) |  |
| **Weight, median [Q1,Q3]** | 78.5 [65.6,94.4] | 79.5 [66.2,95.2] | 74.3 [61.6,88.9] | <0.001 |
| **Weight (leave),median[Q1,Q3]** | 79.5 [66.3,95.4] | 80.0 [66.8,96.2] | 75.1 [63.6,90.5] | <0.001 |
| **BMI, median [Q1,Q3]** | 27.7 [24.0,32.7] | 27.9 [24.2,32.9] | 26.5 [23.0,31.5] | <0.001 |
| **Insurance, n (%)** |  |  |  | <0.001 |
| **Medicaid** | 567 (4.4) | 506 (4.6) | 61 (3.3) |  |
| **Medicare** | 8483 (66.0) | 7112 (64.7) | 1371 (73.5) |  |
| **Other** | 3806 (29.6) | 3372 (30.7) | 434 (23.3) |  |
| **Language, n (%)** |  |  |  | <0.001 |
| **English** | 8080 (62.9) | 7110 (64.7) | 970 (52.0) |  |
| **Other** | 483 (3.8) | 415 (3.8) | 68 (3.6) |  |
| **Unknown** | 4293 (33.4) | 3465 (31.5) | 828 (44.4) |  |
| **Elective surgery, n (%)** | 759 (5.9) | 724 (6.6) | 35 (1.9) | <0.001 |
| **Mechanical ventilation, n (%)** | 5323 (41.4) | 4333 (39.4) | 990 (53.1) | <0.001 |
| **Vasopressor, n (%)** | 3513 (27.3) | 2759 (25.1) | 754 (40.4) | <0.001 |
| **Milrinone, n (%)** | 501 (3.9) | 436 (4.0) | 65 (3.5) | 0.35 |
| **Furosemide, n (%)** | 701 (5.5) | 548 (5.0) | 153 (8.2) | <0.001 |
| **Activity, n (%)** |  |  |  | <0.001 |
| **Bed** | 9374 (73.3) | 7729 (70.6) | 1645 (88.8) |  |
| **Sit** | 2284 (17.8) | 2137 (19.5) | 147 (7.9) |  |
| **Stand** | 1139 (8.9) | 1078 (9.9) | 61 (3.3) |  |
| **Fall risk, n (%)** | 4231 (32.9) | 3557 (32.4) | 674 (36.1) | 0.002 |
| **Braden nutrition, n (%)** |  |  |  | <0.001 |
| **Excellent/Adequate** | 4602 (36.2) | 4293 (39.5) | 309 (16.7) |  |
| **Probably Inadequate** | 7094 (55.8) | 5892 (54.2) | 1202 (65.1) |  |
| **Very Poor** | 1021 (8.0) | 685 (6.3) | 336 (18.2) |  |
| **Braden mobility, n (%)** |  |  |  | <0.001 |
| **Completely Immobile/Very Limited** | 5444 (42.8) | 4198 (38.6) | 1246 (67.5) |  |
| **No/Slight Limitations** | 7275 (57.2) | 6674 (61.4) | 601 (32.5) |  |
| **Braden activity, n (%)** |  |  |  | <0.001 |
| **Bedfast** | 10156 (79.9) | 8476 (78.0) | 1680 (91.0) |  |
| **Chairfast** | 1631 (12.8) | 1521 (14.0) | 110 (6.0) |  |
| **Walks Frequently/Occasionally** | 930 (7.3) | 873 (8.0) | 57 (3.1) |  |
| **LLL Lung sounds, n (%)** |  |  |  | <0.001 |
| **Clear** | 3070 (23.9) | 2765 (25.2) | 305 (16.4) |  |
| **Crackles** | 1855 (14.4) | 1593 (14.5) | 262 (14.0) |  |
| **Diminished/Absent** | 6308 (49.1) | 5410 (49.3) | 898 (48.2) |  |
| **Stridor/others** | 1609 (12.5) | 1209 (11.0) | 400 (21.4) |  |
| **LUL Lung sounds, n (%)** |  |  |  | <0.001 |
| **Clear** | 9217 (71.8) | 8167 (74.4) | 1050 (56.3) |  |
| **Crackles** | 260 (2.0) | 203 (1.8) | 57 (3.1) |  |
| **Diminished/Absent** | 1025 (8.0) | 830 (7.6) | 195 (10.5) |  |
| **Stridor/others** | 2341 (18.2) | 1778 (16.2) | 563 (30.2) |  |
| **RLL Lung sounds, n (%)** |  |  |  | <0.001 |
| **Clear** | 3111 (24.3) | 2802 (25.7) | 309 (16.7) |  |
| **Crackles** | 1889 (14.8) | 1628 (14.9) | 261 (14.1) |  |
| **Diminished/Absent** | 6223 (48.7) | 5321 (48.7) | 902 (48.6) |  |
| **Stridor/others** | 1554 (12.2) | 1171 (10.7) | 383 (20.6) |  |
| **RUL Lung sounds, n (%)** |  |  |  | <0.001 |
| **Clear** | 9267 (72.1) | 8206 (74.7) | 1061 (56.9) |  |
| **Crackles** | 235 (1.8) | 179 (1.6) | 56 (3.0) |  |
| **Diminished/Absent** | 966 (7.5) | 790 (7.2) | 176 (9.4) |  |
| **Stridor/others** | 2377 (18.5) | 1805 (16.4) | 572 (30.7) |  |
| **CCI score, median [Q1,Q3]** | 7.0 [5.0,8.0] | 7.0 [5.0,8.0] | 7.0 [6.0,9.0] | <0.001 |
| **Length of stay before ICU, median [Q1,Q3]** | 0.1 [0.0,1.1] | 0.1 [0.0,1.0] | 0.1 [0.0,1.4] | 0.263 |
| **Length of ICU durations, median [Q1,Q3]** | 3.0 [1.8,5.4] | 2.9 [1.8,5.0] | 4.6 [2.3,8.9] | <0.001 |
| **Length of hospital durations, median [Q1,Q3]** | 8.9 [5.7,14.6] | 8.9 [5.8,14.4] | 8.7 [4.5,15.8] | 0.001 |

**S4 Table. Cohort baseline (discharge hospital: survivor vs. non-survivor within 90 days)**

|  | **Overall**  **(n=10,990)** | **Survivor**  **(n=10,221)** | **Non-survivor**  **(n=769)** | **P-Value** |
| --- | --- | --- | --- | --- |
| **Age, median [Q1,Q3]** | 74 [64,83] | 74 [63,83] | 80 [72,86] | <0.001 |
| **Female, n (%)** | 5054 (46.0) | 4709 (46.1) | 345 (44.9) | 0.541 |
| **Admission type, n (%)** |  |  |  | 0.002 |
| **ELECTIVE** | 913 (8.3) | 872 (8.5) | 41 (5.3) |  |
| **EMERGENCY** | 10077 (91.7) | 9349 (91.5) | 728 (94.7) |  |
| **Ethnicity, n (%)** |  |  |  | 0.004 |
| **Asian** | 220 (2.0) | 210 (2.1) | 10 (1.3) |  |
| **Black** | 1021 (9.3) | 962 (9.4) | 59 (7.7) |  |
| **Hispanic** | 267 (2.4) | 261 (2.6) | 6 (0.8) |  |
| **Other** | 1622 (14.8) | 1498 (14.7) | 124 (16.1) |  |
| **White** | 7860 (71.5) | 7290 (71.3) | 570 (74.1) |  |
| **Marital status, n (%)** |  |  |  | 0.002 |
| **Divorced** | 805 (7.3) | 748 (7.3) | 57 (7.4) |  |
| **Married** | 5083 (46.3) | 4726 (46.2) | 357 (46.4) |  |
| **Unknown** | 2681 (24.4) | 2529 (24.7) | 152 (19.8) |  |
| **Widowed** | 2421 (22.0) | 2218 (21.7) | 203 (26.4) |  |
| **Weight, median [Q1,Q3]** | 79.5 [66.2,95.2] | 80.0 [66.9,96.0] | 71.9 [60.3,85.5] | <0.001 |
| **Weight(leave), median [Q1,Q3]** | 80.0 [66.8,96.2] | 80.7 [67.2,97.0] | 72.6 [60.9,86.2] | <0.001 |
| **BMI, median [Q1,Q3]** | 27.9 [24.2,32.9] | 28.1 [24.4,33.2] | 25.8 [22.4,29.6] | <0.001 |
| **Insurance, n (%)** |  |  |  | <0.001 |
| **Medicaid** | 506 (4.6) | 496 (4.9) | 10 (1.3) |  |
| **Medicare** | 7112 (64.7) | 6475 (63.3) | 637 (82.8) |  |
| **Other** | 3372 (30.7) | 3250 (31.8) | 122 (15.9) |  |
| **Language, n (%)** |  |  |  | <0.001 |
| **English** | 7110 (64.7) | 6863 (67.1) | 247 (32.1) |  |
| **Other** | 415 (3.8) | 355 (3.5) | 60 (7.8) |  |
| **Unknown** | 3465 (31.5) | 3003 (29.4) | 462 (60.1) |  |
| **Elective surgery, n (%)** | 724 (6.6) | 691 (6.8) | 33 (4.3) | 0.010 |
| **Mechanical ventilation, n (%)** | 4333 (39.4) | 4033 (39.5) | 300 (39.0) | 0.837 |
| **Vasopressor, n (%)** | 2759 (25.1) | 2545 (24.9) | 214 (27.8) | 0.078 |
| **Milrinone, n (%)** | 436 (4.0) | 401 (3.9) | 35 (4.6) | 0.444 |
| **Furosemide, n (%)** | 548 (5.0) | 517 (5.1) | 31 (4.0) | 0.240 |
| **Activity, n (%)** |  |  |  | <0.001 |
| **Bed** | 7729 (70.6) | 7099 (69.7) | 630 (82.2) |  |
| **Sit** | 2137 (19.5) | 2044 (20.1) | 93 (12.1) |  |
| **Stand** | 1078 (9.9) | 1035 (10.2) | 43 (5.6) |  |
| **Fall risk, n (%)** | 3557 (32.4) | 3142 (30.7) | 415 (54.0) | <0.001 |
| **Braden nutrition, n (%)** |  |  |  | <0.001 |
| **Excellent/Adequate** | 4293 (39.5) | 4085 (40.4) | 208 (27.5) |  |
| **Probably Inadequate** | 5892 (54.2) | 5414 (53.5) | 478 (63.2) |  |
| **Very Poor** | 685 (6.3) | 615 (6.1) | 70 (9.3) |  |
| **Braden mobility, n (%)** |  |  |  | <0.001 |
| **Completely Immobile/Very Limited** | 4198 (38.6) | 3801 (37.6) | 397 (52.5) |  |
| **No/Slight Limitations** | 6674 (61.4) | 6315 (62.4) | 359 (47.5) |  |
| **Braden activity, n (%)** |  |  |  | <0.001 |
| **Bedfast** | 8476 (78.0) | 7829 (77.4) | 647 (85.6) |  |
| **Chairfast** | 1521 (14.0) | 1449 (14.3) | 72 (9.5) |  |
| **Walks Frequently/Occasionally** | 873 (8.0) | 836 (8.3) | 37 (4.9) |  |
| **LLL Lung sounds, n (%)** |  |  |  | <0.001 |
| **Clear** | 2765 (25.2) | 2588 (25.4) | 177 (23.0) |  |
| **Crackles** | 1593 (14.5) | 1455 (14.3) | 138 (18.0) |  |
| **Diminished/Absent** | 5410 (49.3) | 5077 (49.7) | 333 (43.4) |  |
| **Stridor/others** | 1209 (11.0) | 1089 (10.7) | 120 (15.6) |  |
| **LUL Lung sounds, n (%)** |  |  |  | <0.001 |
| **Clear** | 8167 (74.4) | 7657 (75.0) | 510 (66.4) |  |
| **Crackles** | 203 (1.8) | 186 (1.8) | 17 (2.2) |  |
| **Diminished/Absent** | 830 (7.6) | 772 (7.6) | 58 (7.6) |  |
| **Stridor/others** | 1778 (16.2) | 1595 (15.6) | 183 (23.8) |  |
| **RLL Lung sounds, n (%)** |  |  |  | <0.001 |
| **Clear** | 2802 (25.7) | 2644 (26.0) | 158 (20.6) |  |
| **Crackles** | 1628 (14.9) | 1490 (14.7) | 138 (18.0) |  |
| **Diminished/Absent** | 5321 (48.7) | 4971 (48.9) | 350 (45.7) |  |
| **Stridor/others** | 1171 (10.7) | 1051 (10.3) | 120 (15.7) |  |
| **RUL Lung sounds, n (%)** |  |  |  | <0.001 |
| **Clear** | 8206 (74.7) | 7696 (75.4) | 510 (66.3) |  |
| **Crackles** | 179 (1.6) | 163 (1.6) | 16 (2.1) |  |
| **Diminished/Absent** | 790 (7.2) | 740 (7.2) | 50 (6.5) |  |
| **Stridor/others** | 1805 (16.4) | 1612 (15.8) | 193 (25.1) |  |
| **Mechanical ventilation(leave), n (%)** | 952 (8.7) | 873 (8.5) | 79 (10.3) | 0.114 |
| **Activity (leave), n (%)** |  |  |  | <0.001 |
| **Bed** | 3320 (30.5) | 2956 (29.2) | 364 (47.7) |  |
| **Sit** | 4991 (45.8) | 4668 (46.0) | 323 (42.3) |  |
| **Stand** | 2591 (23.8) | 2515 (24.8) | 76 (10.0) |  |
| **Fall risk(leave), n (%)** | 3908 (35.6) | 3442 (33.7) | 466 (60.6) | <0.001 |
| **Braden nutrition(leave), n (%)** |  |  |  | <0.001 |
| **Excellent/Adequate** | 5776 (54.4) | 5508 (55.7) | 268 (36.7) |  |
| **Probably Inadequate** | 4442 (41.8) | 4037 (40.8) | 405 (55.4) |  |
| **Very Poor** | 397 (3.7) | 339 (3.4) | 58 (7.9) |  |
| **Braden mobility(leave), n (%)** |  |  |  | <0.001 |
| **Completely Immobile/Very Limited** | 2620 (24.7) | 2319 (23.5) | 301 (41.2) |  |
| **No/Slight Limitations** | 7995 (75.3) | 7565 (76.5) | 430 (58.8) |  |
| **Braden activity(leave), n (%)** |  |  |  | <0.001 |
| **Bedfast** | 5033 (47.4) | 4573 (46.3) | 460 (62.9) |  |
| **Chairfast** | 3506 (33.0) | 3304 (33.4) | 202 (27.6) |  |
| **Walks Frequently/Occasionally** | 2075 (19.5) | 2006 (20.3) | 69 (9.4) |  |
| **LLL Lung sounds(leave), n (%)** |  |  |  | <0.001 |
| **Clear** | 3035 (27.7) | 2876 (28.3) | 159 (20.7) |  |
| **Crackles** | 1536 (14.0) | 1413 (13.9) | 123 (16.0) |  |
| **Diminished/Absent** | 5470 (50.0) | 5078 (49.9) | 392 (51.1) |  |
| **Stridor/others** | 898 (8.2) | 805 (7.9) | 93 (12.1) |  |
| **LUL Lung sounds(leave), n (%)** |  |  |  | <0.001 |
| **Clear** | 8783 (80.3) | 8235 (80.9) | 548 (71.4) |  |
| **Crackles** | 137 (1.3) | 129 (1.3) | 8 (1.0) |  |
| **Diminished/Absent** | 742 (6.8) | 678 (6.7) | 64 (8.3) |  |
| **Stridor/others** | 1279 (11.7) | 1132 (11.1) | 147 (19.2) |  |
| **RLL Lung sounds(leave), n (%)** |  |  |  | <0.001 |
| **Clear** | 3042 (27.9) | 2889 (28.6) | 153 (19.9) |  |
| **Crackles** | 1523 (14.0) | 1396 (13.8) | 127 (16.5) |  |
| **Diminished/Absent** | 5443 (50.0) | 5056 (50.0) | 387 (50.4) |  |
| **Stridor/others** | 879 (8.1) | 778 (7.7) | 101 (13.2) |  |
| **RUL Lung sounds(leave), n (%)** |  |  |  | <0.001 |
| **Clear** | 8814 (80.5) | 8262 (81.2) | 552 (71.9) |  |
| **Crackles** | 117 (1.1) | 110 (1.1) | 7 (0.9) |  |
| **Diminished/Absent** | 689 (6.3) | 632 (6.2) | 57 (7.4) |  |
| **Stridor/others** | 1323 (12.1) | 1171 (11.5) | 152 (19.8) |  |
| **CCI score, median [Q1,Q3]** | 7.0 [5.0,8.0] | 7.0 [5.0,8.0] | 7.0 [6.0,9.0] | <0.001 |
| **Length of stay before ICU, median [Q1,Q3]** | 0.1 [0.0,1.0] | 0.1 [0.0,1.0] | 0.1 [0.0,1.7] | 0.013 |
| **Length of ICU durations, median [Q1,Q3]** | 2.9 [1.8,5.0] | 2.8 [1.8,4.9] | 3.6 [2.1,7.0] | <0.001 |
| **Length of hospital durations, median [Q1,Q3]** | 8.9 [5.8,14.4] | 8.8 [5.7,14.0] | 11.9 [7.0,18.8] | <0.001 |

**S5 Table. Cohort baseline (discharge hospital: survivor vs. non-survivor within 1 year)**

|  | **Overall**  **(n=10221)** | **Survivor**  **(n=9509)** | **Non-survivor**  **(n=1481)** | **P-Value** |
| --- | --- | --- | --- | --- |
| **Age, median [Q1,Q3]** | 74 [64,83] | 73 [63,83] | 78 [70,85] | <0.001 |
| **Female, n (%)** | 5054 (46.0) | 4367 (45.9) | 687 (46.4) | 0.761 |
| **Admission type, n (%)** |  |  |  | 0.005 |
| **ELECTIVE** | 913 (8.3) | 818 (8.6) | 95 (6.4) |  |
| **EMERGENCY** | 10077 (91.7) | 8691 (91.4) | 1386 (93.6) |  |
| **Ethnicity, n (%)** |  |  |  | 0.016 |
| **Asian** | 220 (2.0) | 194 (2.0) | 26 (1.8) |  |
| **Black** | 1021 (9.3) | 893 (9.4) | 128 (8.6) |  |
| **Hispanic** | 267 (2.4) | 248 (2.6) | 19 (1.3) |  |
| **Other** | 1622 (14.8) | 1387 (14.6) | 235 (15.9) |  |
| **White** | 7860 (71.5) | 6787 (71.4) | 1073 (72.5) |  |
| **Marital status, n (%)** |  |  |  | <0.001 |
| **Divorced** | 805 (7.3) | 688 (7.2) | 117 (7.9) |  |
| **Married** | 5083 (46.3) | 4406 (46.3) | 677 (45.7) |  |
| **Unknown** | 2681 (24.4) | 2376 (25.0) | 305 (20.6) |  |
| **Widowed** | 2421 (22.0) | 2039 (21.4) | 382 (25.8) |  |
| **Weight, median [Q1,Q3]** | 79.5 [66.2,95.2] | 80.0 [67.2,96.3] | 73.0 [61.4,87.5] | <0.001 |
| **Weight(leave), median [Q1,Q3]** | 80.0 [66.8,96.2] | 81.0 [67.6,97.3] | 74.0 [62.0,88.1] | <0.001 |
| **BMI, median [Q1,Q3]** | 27.9 [24.2,32.9] | 28.2 [24.6,33.3] | 26.1 [22.5,30.2] | <0.001 |
| **Insurance, n (%)** |  |  |  | <0.001 |
| **Medicaid** | 506 (4.6) | 463 (4.9) | 43 (2.9) |  |
| **Medicare** | 7112 (64.7) | 5919 (62.2) | 1193 (80.6) |  |
| **Other** | 3372 (30.7) | 3127 (32.9) | 245 (16.5) |  |
| **Language, n (%)** |  |  |  | <0.001 |
| **English** | 7110 (64.7) | 6657 (70.0) | 453 (30.6) |  |
| **Other** | 415 (3.8) | 299 (3.1) | 116 (7.8) |  |
| **Unknown** | 3465 (31.5) | 2553 (26.8) | 912 (61.6) |  |
| **Elective surgery, n (%)** | 724 (6.6) | 651 (6.8) | 73 (4.9) | 0.007 |
| **Mechanical ventilation, n (%)** | 4333 (39.4) | 3739 (39.3) | 594 (40.1) | 0.584 |
| **Vasopressor, n (%)** | 2759 (25.1) | 2360 (24.8) | 399 (26.9) | 0.085 |
| **Milrinone, n (%)** | 436 (4.0) | 381 (4.0) | 55 (3.7) | 0.641 |
| **Furosemide, n (%)** | 548 (5.0) | 481 (5.1) | 67 (4.5) | 0.415 |
| **Activity, n (%)** |  |  |  | <0.001 |
| **Bed** | 7729 (70.6) | 6538 (69.0) | 1191 (80.7) |  |
| **Sit** | 2137 (19.5) | 1944 (20.5) | 193 (13.1) |  |
| **Stand** | 1078 (9.9) | 987 (10.4) | 91 (6.2) |  |
| **Fall risk, n (%)** | 3557 (32.4) | 2756 (29.0) | 801 (54.1) | <0.001 |
| **Braden nutrition, n (%)** |  |  |  | <0.001 |
| **Excellent/Adequate** | 4293 (39.5) | 3855 (41.0) | 438 (30.1) |  |
| **Probably Inadequate** | 5892 (54.2) | 4990 (53.0) | 902 (61.9) |  |
| **Very Poor** | 685 (6.3) | 568 (6.0) | 117 (8.0) |  |
| **Braden mobility, n (%)** |  |  |  | <0.001 |
| **Completely Immobile/Very Limited** | 4198 (38.6) | 3486 (37.0) | 712 (48.8) |  |
| **No/Slight Limitations** | 6674 (61.4) | 5928 (63.0) | 746 (51.2) |  |
| **Braden activity, n (%)** |  |  |  | <0.001 |
| **Bedfast** | 8476 (78.0) | 7232 (76.8) | 1244 (85.4) |  |
| **Chairfast** | 1521 (14.0) | 1385 (14.7) | 136 (9.3) |  |
| **Walks Frequently/Occasionally** | 873 (8.0) | 796 (8.5) | 77 (5.3) |  |
| **LLL Lung sounds, n (%)** |  |  |  | <0.001 |
| **Clear** | 2765 (25.2) | 2423 (25.5) | 342 (23.1) |  |
| **Crackles** | 1593 (14.5) | 1318 (13.9) | 275 (18.6) |  |
| **Diminished/Absent** | 5410 (49.3) | 4784 (50.4) | 626 (42.4) |  |
| **Stridor/others** | 1209 (11.0) | 974 (10.3) | 235 (15.9) |  |
| **LUL Lung sounds, n (%)** |  |  |  | <0.001 |
| **Clear** | 8167 (74.4) | 7156 (75.3) | 1011 (68.4) |  |
| **Crackles** | 203 (1.8) | 176 (1.9) | 27 (1.8) |  |
| **Diminished/Absent** | 830 (7.6) | 734 (7.7) | 96 (6.5) |  |
| **Stridor/others** | 1778 (16.2) | 1434 (15.1) | 344 (23.3) |  |
| **RLL Lung sounds, n (%)** |  |  |  | <0.001 |
| **Clear** | 2802 (25.7) | 2467 (26.1) | 335 (22.7) |  |
| **Crackles** | 1628 (14.9) | 1360 (14.4) | 268 (18.2) |  |
| **Diminished/Absent** | 5321 (48.7) | 4679 (49.5) | 642 (43.5) |  |
| **Stridor/others** | 1171 (10.7) | 940 (10.0) | 231 (15.7) |  |
| **RUL Lung sounds, n (%)** |  |  |  | <0.001 |
| **Clear** | 8206 (74.7) | 7188 (75.7) | 1018 (68.8) |  |
| **Crackles** | 179 (1.6) | 155 (1.6) | 24 (1.6) |  |
| **Diminished/Absent** | 790 (7.2) | 706 (7.4) | 84 (5.7) |  |
| **Stridor/others** | 1805 (16.4) | 1451 (15.3) | 354 (23.9) |  |
| **Mechanical ventilation(leave), n (%)** | 952 (8.7) | 820 (8.6) | 132 (8.9) | 0.750 |
| **Activity(leave), n (%)** |  |  |  | <0.001 |
| **Bed** | 3320 (30.5) | 2656 (28.2) | 664 (45.3) |  |
| **Sit** | 4991 (45.8) | 4370 (46.3) | 621 (42.3) |  |
| **Stand** | 2591 (23.8) | 2409 (25.5) | 182 (12.4) |  |
| **Fall risk(leave), n (%)** | 3908 (35.6) | 3003 (31.6) | 905 (61.1) | <0.001 |
| **Braden nutrition(leave), n (%)** |  |  |  | <0.001 |
| **Excellent/Adequate** | 5776 (54.4) | 5191 (56.4) | 585 (41.7) |  |
| **Probably Inadequate** | 4442 (41.8) | 3704 (40.2) | 738 (52.6) |  |
| **Very Poor** | 397 (3.7) | 317 (3.4) | 80 (5.7) |  |
| **Braden mobility(leave), n (%)** |  |  |  | <0.001 |
| **Completely Immobile/Very Limited** | 2620 (24.7) | 2098 (22.8) | 522 (37.2) |  |
| **No/Slight Limitations** | 7995 (75.3) | 7114 (77.2) | 881 (62.8) |  |
| **Braden activity(leave), n (%)** |  |  |  | <0.001 |
| **Bedfast** | 5033 (47.4) | 4176 (45.3) | 857 (61.1) |  |
| **Chairfast** | 3506 (33.0) | 3118 (33.9) | 388 (27.7) |  |
| **Walks Frequently/Occasionally** | 2075 (19.5) | 1917 (20.8) | 158 (11.3) |  |
| **LLL Lung sounds(leave), n (%)** |  |  |  | <0.001 |
| **Clear** | 3035 (27.7) | 2695 (28.5) | 340 (23.1) |  |
| **Crackles** | 1536 (14.0) | 1278 (13.5) | 258 (17.5) |  |
| **Diminished/Absent** | 5470 (50.0) | 4784 (50.5) | 686 (46.5) |  |
| **Stridor/others** | 898 (8.2) | 707 (7.5) | 191 (12.9) |  |
| **LUL Lung sounds(leave), n (%)** |  |  |  | 0.001 |
| **Clear** | 8783 (80.3) | 7684 (81.2) | 1099 (74.5) |  |
| **Crackles** | 137 (1.3) | 119 (1.3) | 18 (1.2) |  |
| **Diminished/Absent** | 742 (6.8) | 640 (6.8) | 102 (6.9) |  |
| **Stridor/others** | 1279 (11.7) | 1023 (10.8) | 256 (17.4) |  |
| **RLL Lung sounds(leave), n (%)** |  |  |  | <0.001 |
| **Clear** | 3042 (27.9) | 2703 (28.7) | 339 (23.0) |  |
| **Crackles** | 1523 (14.0) | 1272 (13.5) | 251 (17.0) |  |
| **Diminished/Absent** | 5443 (50.0) | 4763 (50.6) | 680 (46.1) |  |
| **Stridor/others** | 879 (8.1) | 673 (7.2) | 206 (14.0) |  |
| **RUL Lung sounds(leave), n (%)** |  |  |  | 0.001 |
| **Clear** | 8814 (80.5) | 7704 (81.4) | 1110 (75.2) |  |
| **Crackles** | 117 (1.1) | 106 (1.1) | 11 (0.7) |  |
| **Diminished/Absent** | 689 (6.3) | 597 (6.3) | 92 (6.2) |  |
| **Stridor/others** | 1323 (12.1) | 1059 (11.2) | 264 (17.9) |  |
| **CCI score, median [Q1,Q3]** | 7.0 [5.0,8.0] | 7.0 [5.0,8.0] | 7.0 [6.0,9.0] | <0.001 |
| **Length of stay before ICU, median [Q1,Q3]** | 0.1 [0.0,1.0] | 0.1 [0.0,1.0] | 0.1 [0.0,1.5] | <0.001 |
| **Length of ICU durations, median [Q1,Q3]** | 2.9 [1.8,5.0] | 2.8 [1.8,4.9] | 3.3 [2.0,6.2] | <0.001 |
| **Length of hospital durations, median [Q1,Q3]** | 8.9 [5.8,14.4] | 8.7 [5.7,13.9] | 11.2 [6.8,18.0] | <0.001 |

**S6 Table. Detailed model performance for short-term outcomes of internal test set and external test set (95% CI)**

|  | Model | AUROC | Sensitivity | Specificity | Accuracy | F1 score | Precision | AUPRC |
| --- | --- | --- | --- | --- | --- | --- | --- | --- |
| In-hospital mortality | Internal test set (mimic) | **0.836 (0.831-0.844)** | **0.761 (0.694-0.813)** | **0.748 (0.695-0.807)** | **0.750 (0.711-0.793)** | **0.454 (0.435-0.482)** | **0.325 (0.301-0.366)** | **0.279 (0.264-0.299)** |
|  | External test set (eicu) | 0.769 (0.765-0.773) | 0.707 (0.699-0.716) | 0.703 (0.699-0.712) | 0.704 (0.700-0.710) | 0.418 (0.412-0.423) | 0.296 (0.291-0.301) | 0.254 (0.249-0.258) |

**S7 Table. Features ranking of short- and long-term outcome prediction**

| **Ranking** | **Features** | | |
| --- | --- | --- | --- |
|  | **In-hospital mortality** | **90-day mortality** | **1-year mortality** |
| **1** | GCS(min) | Age | Fall risk(leave) |
| **2** | Urine output | BUN(max)(leave) | Primary language |
| **3** | Braden mobility | Fall risk(leave) | Urine output(leave) |
| **4** | BUN(max) | Primary language | Temperature(mean)(leave) |
| **5** | Respiratory rate(mean) | Temperature(mean)(leave) | CCI score |
| **6** | Age | Urine output(leave) | BUN(max)(leave) |
| **7** | Heart rate(mean) | BUN(max) | BUN(max) |
| **8** | CCI score | Urine output | Age |
| **9** | SBP(mean) | SBP(mean)(leave) | Creatinine(max) |
| **10** | Weight | Respiratory rate(mean)(leave) | Urine output |
| **11** | Braden nutrition | Insurance | Respiratory rate(mean)(leave) |
| **12** | Activity | CCI score | SBP(mean)(leave) |
| **13** | Primary language | WBC(max)(leave) | Insurance |
| **14** | PaO2(min) | Albumin(min) | BMI |
| **15** | Aniongap(max) | Weight | Fall risk |
| **16** | INR(min) | PTT(max)(leave) | Heart rate(mean)(leave) |
| **17** | PTT(max) | Weight(leave) | Chloride(min) |
| **18** | RUL Lung sounds | Temperature(mean) | preICU LOS day |
| **19** | SpO2(min) | Platelet(min) | Weight(leave) |
| **20** | PaCO2(max) | preICU LOS day | Platelet(min) |
| **21** | Bilirubin(max) | BMI | PT(max) |
| **22** | ALP(max) | PT(max) | Weight |
| **23** | Glucose(max) | Respiratory rate(mean) | WBC(max) |
| **24** | SpO2/FiO2 | Heart rate(mean)(leave) | Temperature(mean) |
| **25** | Platelet(min) | Platelet(min)(leave) | PTT(max)(leave) |
| **26** | preICU LOS day | Activity(leave) | Activity(leave) |
| **27** | Troponin(max) | Braden nutrition(leave) | Hematocrit(max) |
| **28** | Potassium(max) | Hematocrit(max) | Gender |
| **29** | Hematocrit(max) | SBP(mean) | Hemoglobin(min)(leave) |
| **30** | Albumin(min) | WBC(max) | WBC(max)(leave) |
| **31** | BMI | Creatinine(max) | Respiratory rate(mean) |
| **32** | LUL Lung sounds | Sodium(max) | Heart rate(mean) |
| **33** | Temperature(mean) | Hemoglobin(min)(leave) | Platelet(min)(leave) |
| **34** | Creatinine(max) | Heart rate(mean) | SBP(mean) |
| **35** | MBP(mean) | ALP(max) | Weight change |
| **36** | Hemoglobin(min) | Fall risk | MBP(mean) |
| **37** | Lactate(max) | Hemoglobin(min) | Troponin(max) |
| **38** | AST(max) | Braden mobility(leave) | PaCO2(max) |
| **39** | Sodium(max) | Gender | Hemoglobin(min) |
| **40** | Lymphocytes(min) | Potassium(max)(leave) | Hematocrit(max)(leave) |
| **41** | Chloride(min) | MBP(mean)(leave) | PTT(max) |
| **42** | PT(max) | MBP(mean) | Sodium(max) |
| **43** | Ventilation | ALT(max) | ALP(max) |
| **44** | Lymphocytes(max) | PTT(max) | Glucose(max) |
| **45** | WBC(max) | Glucose(max)(leave) | PT(max)(leave) |
| **46** | Ethnicity | Glucose(max) | GCS(min)(leave) |
| **47** | Magnesium(max) | RUL Lung sounds | Braden mobility(leave) |
| **48** | BaseExcess(min) | SpO2(min)(leave) | Braden nutrition(leave) |
| **49** | Neutrophils(min) | Hematocrit(max)(leave) | Albumin(min) |
| **50** | Bicarbonate(min) | Neutrophils(min) | MBP(mean)(leave) |
| **51** | Elective surgery | SpO2/FiO2 | SpO2/FiO2 |
| **52** | RLL Lung sounds | Potassium(max) | troponin_max_eva_flag |
| **53** | ALT(max) | INR(min) | ALT(max) |
| **54** | Braden activity | Aniongap(max) | INR(min) |
| **55** | Bicarbonate(max) | Weight change | Potassium(max) |
| **56** | Fall risk | Bicarbonate(max)(leave) | Bicarbonate(max) |
| **57** | alt_max_eva_flag | Chloride(min) | SpO2(min)(leave) |
| **58** | LLL Lung sounds | Chloride(min)(leave) | Potassium(max)(leave) |
| **59** | pao2_min_eva_flag | Troponin(max) | GCS(min) |
| **60** | troponin_max_eva_flag | PT(max)(leave) | Braden activity(leave) |
| **61** | Vasopressor | Bicarbonate(min) | Glucose(max)(leave) |
| **62** | Furosemide | INR(min)(leave) | INR(min)(leave) |
| **63** | bilirubin_max_eva_flag | Creatinine(max)(leave) | SpO2(min) |
| **64** | Insurance | RLL Lung sounds | RUL Lung sounds |
| **65** | Marital status | PaO2(min) | Sodium(max)(leave) |
| **66** | ast_max_eva_flag | Lymphocytes(max) | Aniongap(max) |
| **67** | Admission type | Magnesium(max) | FiO2(max) |
| **68** | FiO2(max) | Magnesium(max)(leave) | Creatinine(max)(leave) |
| **69** | alp_max_eva_flag | RLL Lung sounds(leave) | Lymphocytes(max) |
| **70** | neutrophils_min_eva_flag | PaCO2(max) | Lymphocytes(min) |
| **71** | albumin_min_eva_flag | Bicarbonate(min)(leave) | PaO2(min) |
| **72** | lactate_max_eva_flag | SpO2(min) | Chloride(min)(leave) |
| **73** | paco2_max_eva_flag | Braden mobility | Neutrophils(min) |
| **74** | Gender | GCS(min)(leave) | Bicarbonate(min) |
| **75** | lymphocytes_max_eva_flag | Bicarbonate(max) | ptt_max_leave_eva_flag |
| **76** | Milrinone | AST(max) | Lactate(max) |
| **77** | fio2_max_eva_flag | Braden activity(leave) | RLL Lung sounds(leave) |
| **78** | lymphocytes_min_eva_flag | GCS(min) | Bicarbonate(max)(leave) |
| **79** | baseexcess_min_eva_flag | BaseExcess(min) | AST(max) |
| **80** |  | Lymphocytes(min) | Magnesium(max) |
| **81** |  | Sodium(max)(leave) | Bicarbonate(min)(leave) |
| **82** |  | Aniongap(max)(leave) | Magnesium(max)(leave) |
| **83** |  | Bilirubin(max) | RLL Lung sounds |
| **84** |  | Lactate(max) | Bilirubin(max) |
| **85** |  | RUL Lung sounds(leave) | LUL Lung sounds |
| **86** |  | Activity | BaseExcess(min) |
| **87** |  | LLL Lung sounds(leave) | Ventilation |
| **88** |  | Braden nutrition | Aniongap(max)(leave) |
| **89** |  | LUL Lung sounds(leave) | lymphocytes_max_eva_flag |
| **90** |  | LUL Lung sounds | Activity |
| **91** |  | Marital | Ethnicity |
| **92** |  | Ventilation | pt_max_leave_eva_flag |
| **93** |  | ptt_max_leave_eva_flag | LLL Lung sounds(leave) |
| **94** |  | FiO2(max) | Marital |
| **95** |  | LLL Lung sounds | Braden mobility |
| **96** |  | lymphocytes_max_eva_flag | LLL Lung sounds |
| **97** |  | Furosemide | Braden nutrition |
| **98** |  | pt_max_leave_eva_flag | LUL Lung sounds(leave) |
| **99** |  | Admission type | Admission type |
| **100** |  | albumin_min_eva_flag | Braden activity |
| **101** |  | Ventilation(leave) | RUL Lung sounds(leave) |
| **102** |  | alt_max_eva_flag | Vasopressor |
| **103** |  | Ethnicity | ast_max_eva_flag |
| **104** |  | troponin_max_eva_flag | alt_max_eva_flag |
| **105** |  | lymphocytes_min_eva_flag | Ventilation(leave) |
| **106** |  | lactate_max_eva_flag | lactate_max_eva_flag |
| **107** |  | pao2_min_eva_flag | lymphocytes_min_eva_flag |
| **108** |  | Vasopressor | neutrophils_min_eva_flag |
| **109** |  | Braden activity | pao2_min_eva_flag |
| **110** |  | Elective surgery | albumin_min_eva_flag |
| **111** |  | inr_min_leave_eva_flag | fio2_max_eva_flag |
| **112** |  | bilirubin_max_eva_flag | Furosemide |
| **113** |  | alp_max_eva_flag | alp_max_eva_flag |
| **114** |  | neutrophils_min_eva_flag | inr_min_leave_eva_flag |
| **115** |  | furosemide_leave | furosemide_leave |
| **116** |  | baseexcess_min_eva_flag | bilirubin_max_eva_flag |
| **117** |  | ast_max_eva_flag | Elective surgery |
| **118** |  | Milrinone | vasopressor_leave |
| **119** |  | vasopressor_leave | baseexcess_min_eva_flag |
| **120** |  | paco2_max_eva_flag | paco2_max_eva_flag |
| **121** |  | fio2_max_eva_flag | Milrinone |
| **122** |  | milrinone_leave | milrinone_leave |

**S8 Table. Comparison of the models’ performance incorporating with SDOH, physical frailty and lung sounds**

|  | **In-hospital** | | **90-day mortality** | | **1-year mortality** | |
| --- | --- | --- | --- | --- | --- | --- |
| **Features** | **AUROC** | **Variety** | **AUROC** | **Variety** | **AUROC** | **Variety** |
| All | 0.836 | - | 0.790 | - | 0.789 | - |
| Insurance | 0.832 | -0.004 | 0.780 | -0.010 | 0.784 | -0.005 |
| Primary Language | 0.833 | -0.003 | 0.771 | -0.019 | 0.779 | -0.010 |
| Fall risk | 0.835 | -0.001 | 0.773 | -0.017 | 0.780 | -0.009 |
| Activity | 0.833 | -0.003 | 0.779 | -0.011 | 0.785 | -0.004 |
| Braden nutrition | 0.835 | -0.001 | 0.772 | -0.018 | 0.785 | -0.004 |
| Braden mobility | 0.836 | - | 0.781 | -0.009 | 0.785 | -0.004 |
| Braden activity | 0.834 | -0.002 | 0.780 | -0.010 | 0.783 | -0.006 |
| LLL lung sounds | 0.834 | -0.002 | 0.782 | -0.008 | 0.786 | -0.003 |
| LUL lung sounds | 0.834 | -0.002 | 0.781 | -0.009 | 0.783 | -0.006 |
| RLL lung sounds | 0.834 | -0.002 | 0.782 | -0.008 | 0.785 | -0.004 |
| RUL lung sounds | 0.836 | - | 0.779 | -0.011 | 0.787 | -0.002 |

*Note: Variety = AUROC_drop feature_ - AUROC_all_*

**S9 Table. The performance changing of including part of features (AUROC)**

| Feature number | In-hospital | 90-day | 1-year |
| --- | --- | --- | --- |
| 5 | 0.715 | 0.658 | 0.696 |
| 10 | 0.753 | 0.662 | 0.681 |
| 15 | 0.782 | 0.652 | 0.683 |
| 20 | 0.789 | 0.697 | 0.707 |
| 25 | 0.791 | 0.702 | 0.708 |
| 30 | 0.797 | 0.681 | 0.726 |
| 35 | 0.791 | 0.695 | 0.739 |
| 40 | 0.798 | 0.694 | 0.735 |
| 45 | 0.810 | 0.707 | 0.742 |
| 50 | 0.806 | 0.724 | 0.734 |
| 55 | 0.815 | 0.716 | 0.743 |
| 60 | 0.811 | 0.718 | 0.751 |
| 65 | 0.804 | 0.716 | 0.757 |
| 70 | 0.818 | 0.721 | 0.752 |
| 75 | 0.808 | 0.719 | 0.752 |
| 80 |  | 0.725 | 0.763 |
| 85 |  | 0.715 | 0.763 |
| 90 |  | 0.721 | 0.784 |
| 95 |  | 0.755 | 0.778 |
| 100 |  | 0.734 | 0.790 |
| 105 |  | 0.781 | 0.783 |
| 110 |  | 0.770 | 0.790 |
| 115 |  | 0.758 | 0.781 |
| 120 |  | 0.759 | 0.787 |

**S10 Table. Comparison of the models’ performance incorporating with data measured at the first and last day in ICU**

|  | Model | Data measured at the first day in ICU | Data measured at the last day in ICU | Two days included data |
| --- | --- | --- | --- | --- |
| 90-day mortality | XGB | 0.765 [0.756-0.775] | 0.779 [0.766-0.791] | 0.790 [0.780-0.800] |
| 1-year mortality | XGB | 0.775 [0.767-0.784] | 0.786 [0.777-0.794] | 0.789 [0.780-0.799] |


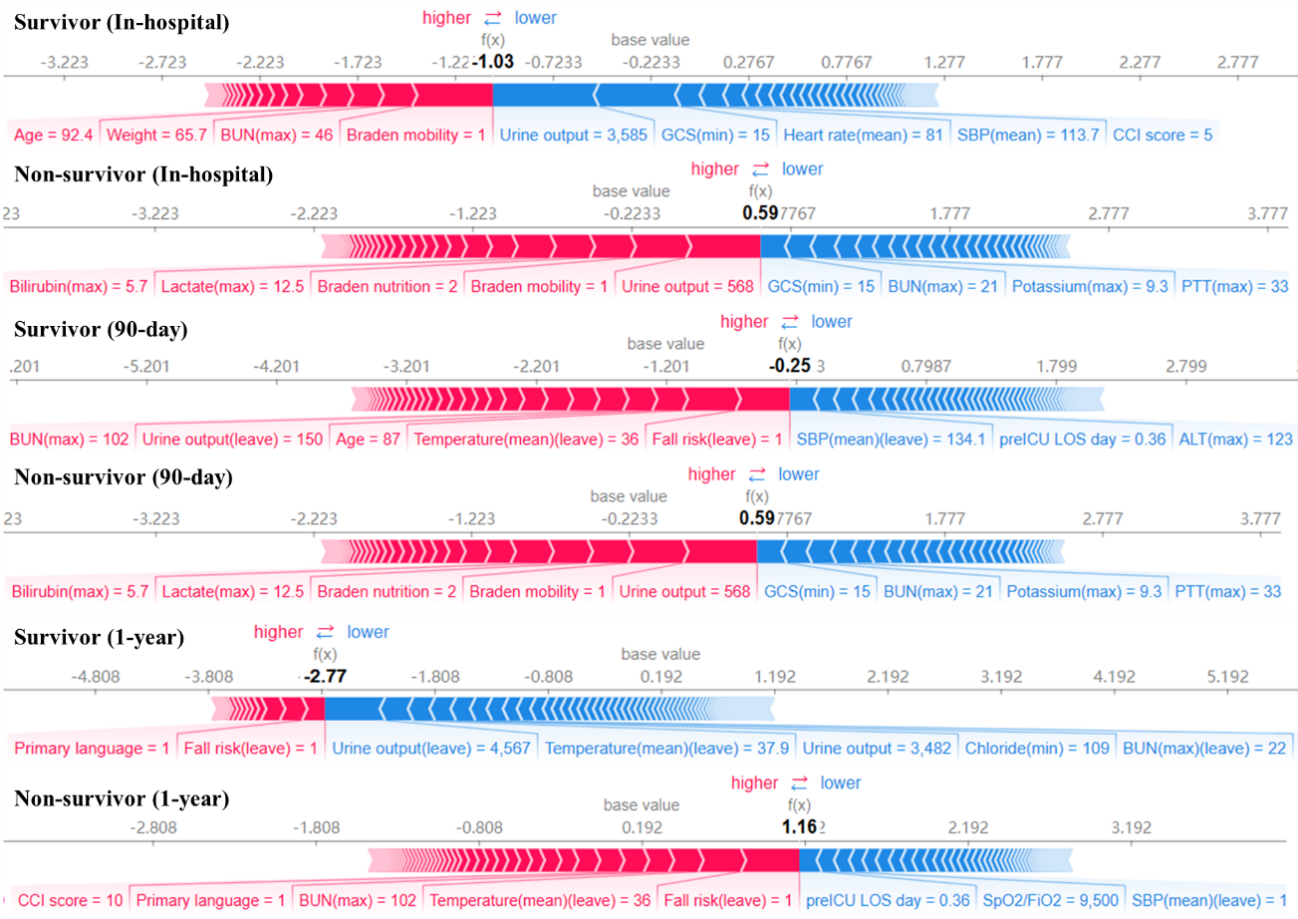


**S1 Fig. The interpretation of in-hospital, 90-day, and 1-year mortality risk prediction models (non-survivor and survivor cases)**
