# Supplementary material for: Explainable mortality prediction models incorporating social health determinants and physical frailty for heart failure patients
Source: PLoS One. 2025 Sep 3;20(9):e0327979. doi: 10.1371/journal.pone.0327979 (PMC12407480; doi:10.1371/journal.pone.0327979)
Supplement: Attachment — Submitted filename: response to reviewers.docx [file pone.0327979.s002.docx]

**Responses to Reviewers’ Comments**

First of all, we would like to express our sincere gratitude to the editor and reviewers for their huge effort in giving excellent suggestions that would significantly improve the quality and readability of this manuscript for revising. We have studied all the comments carefully and have made corrections which we hope meet with approval.

**Responses to Reviewer #1**

**Comment 1**:

What is the reason for limiting to only 4 SDOH factors? There has been research that associated SDOH factors such socioeconomic status, and education or health literacy to HF. Why were these factors not considered?

Response:

Thank you for your insightful question. The decision to limit our study to 2 not 4 Social Determinants of Health (SDOH) factors was based on several considerations. First, we focused on factors that have been consistently shown to have a significant and direct impact on heart failure outcomes, include insurance status and language proficiency. These factors were selected because they are more readily quantifiable and have robust data available in the datasets we utilized.

Our goal was to create a model that is both explainable and clinically applicable. By focusing on a smaller, more targeted set of SDOH factors, we aimed to enhance the interpretability of our model's predictions, ensuring that it remains a practical tool for healthcare providers managing HF patients. However, we recognize that future studies could explore the inclusion of a broader range of SDOH factors, especially in datasets where these variables are well-represented and standardized.

**Comment 2**:

2. Majority of the prior work is discussed in the Discussion section which needs to be moved to the Introduction for better organization and clarity. There are prior studies that explored SDOH and 90-day mortality in HF patients (see below) which needs to be compared with the current work.

Sterling MR, Ringel JB, Pinheiro LC, Safford MM, Levitan EB, Phillips E, Brown TM, Goyal P. Social determinants of health and 90?day mortality after hospitalization for heart failure in the REGARDS study. Journal of the American Heart Association. 2020 May 5;9(9):e014836.

Response:

We appreciate the feedback regarding the organization of the manuscript. We will move the discussion of the majority of prior work from the Discussion section to the Introduction to enhance clarity and provide a better context for our study.

Additionally, we acknowledge the importance of comparing our findings with previous studies that have explored the impact of Social Determinants of Health (SDOH) on 90-day mortality in heart failure patients. We will include a comparison with the study by Sterling et al. (2020), as referenced, in the revised manuscript. This study provides valuable insights, and we will ensure it is appropriately cited and discussed in the context of our findings.

**Comment 3**:

3. The objective of the paper as mentioned in the abstract and conclusion is to develop and validate ML models. Hence it is expected to show these results in the main manuscript and not in the supplements. The authors need to analyze their objectives and main findings and move relevant tables to the main manuscript.

Response:

We understand the importance of aligning the presentation of our results with the stated objectives of the paper. Given that the development and validation of the machine learning models are central to our study, we agree that these results should be highlighted in the main manuscript rather than the supplementary materials.

We will revise the manuscript to move the relevant tables and analyses from the supplements to the main body of the paper. This will ensure that the main findings and their implications are clearly presented and easily accessible to the reader, in alignment with our research objectives.

**Comment 4**:

4. “As shown, our prediction models consistently outperformed all models and scores.” I am not sure what scores is referred to here. If this is about the 7 metrics in eTable6, LR is consistently better for specificity, accuracy, F1, etc.

Response:

Thank you for pointing this out. The reference to "scores" was intended to indicate model performance metrics, specifically the Area Under the Curve (AUC). We apologize for any confusion. Our prediction models consistently outperformed the other models in terms of AUC, which was the primary metric we used to evaluate performance. We will clarify this in the manuscript to avoid any ambiguity and ensure that the comparison is accurately represented.

**Comment 5**:

5. “Representative statistical features were calculated based on the type of variable, such as the maximum, minimum, mean, minus, ..” what does minus mean? Are you referring to the difference between the values?

Response:

Thank you for your question. Yes, "minus" refers to the difference between specific values, such as the difference between the maximum and minimum values or between consecutive observations. We will clarify this term in the manuscript to make the intended meaning more explicit.

**Comment 6**:

6. “We found both of them could improve the discrimination …” what do you mean by “both of them”? It is not clear from the context.

Response:

Thank you for pointing that out. The phrase "both of them" was intended to refer to the all the factors in the fully model, including SDOH (insurance and primary language), PF (fall risk, activity, Braden nutrition, Braden mobility, Braden activity) and LS (LLL, LUL, RLL, and RUL). We apologize for the lack of clarity. We will revise the sentence to explicitly state what "both of them" refers to, ensuring the context is clear to the reader.

**Comment 7**:

7. “The potential bias and injustice of HF patients faced by race in care and outcomes has recently been recognized by researchers, and they have been undertaken to mitigate this dual social and biological impact by building the model separately or incorporate race into the model” – This sentence is highly ambiguous and complex. It is better to split this and explain in detail. Also, references are missing.

Response:

We agree that the sentence could be clearer and more detailed. We will revise it as follows:

Recent research has highlighted the potential bias and disparities faced by heart failure (HF) patients based on race, impacting both care and outcomes. Researchers have recognized this dual social and biological impact and have undertaken efforts to address it. Some approaches involve building separate models for different racial groups to account for these disparities, while others incorporate race directly into the models to mitigate the impact of racial bias.

We will also include relevant references to support this discussion and provide more context in the revised manuscript.

**Comment 8**:

8. “Survival curves in Figure 1(c) (e) show that survival rates are lower for patients with health insurance and older patients, respectively.” Recheck the statement and order of the figure.

Response:

Thank you for your observation. We have rechecked the statement and the order of the figures. Our results are consistent with established understanding: older patients generally have lower survival rates, and in the context of U.S. healthcare, patients with Medicare tend to have lower survival rates compared to those with other types of insurance.

We will ensure that the figure labels and descriptions accurately reflect these findings and make any necessary adjustments to clarify this in the manuscript.

**Comment 9**:

9. Some sentences need to be rephrased as they only convey partial information and is not a complete sentence.

e.g., “While most related studies are limited to small cohort sizes.”

Response:

Thank you for pointing this out. We will revise the incomplete sentences to ensure they convey complete and clear information. For example, the sentence "While most related studies are limited to small cohort sizes" can be rephrased as: "Many related studies are limited by small cohort sizes, which can affect the generalizability and robustness of their findings."

We will review the manuscript to identify and correct any other incomplete sentences to improve clarity and completeness.

**Comment 10**:

10. Many references appear as “ Error! Reference source not found”

Response:

Thank you for bringing this to our attention. We will correct the issue with the references appearing as “Error! Reference source not found.” Our team will review and update the reference list to ensure that all citations are properly linked and formatted correctly in the revised manuscript.

**Comment 11**:

11. Some typos – SODH, martial (should be marital)

Response:

Thank you for pointing out these typographical errors. We will correct the typos, including "SODH" to "SDOH" and "martial" to "marital," in the revised manuscript. We appreciate your attention to detail.

**Comment 12**:

12. Introduction – “require admitted to intensive care unit (ICU)” – should be "admission" instead of admitted

Response:

Thank you for noting that. We will correct the sentence to use "admission" instead of "admitted" in the Introduction section to ensure proper phrasing.

**Comment 13**:

13. “patients, which has been demonstrated high” – should be " ... which has demonstrated ..."

Response:

Thank you for the correction. We will revise the sentence to say, "... which has demonstrated high ..." to ensure proper grammar and clarity.

**Responses to Reviewer #2**

**Comment 1**:

what is the reason of using 90-days and 1-year intervals, literature or stat justification is required.

Response:

Thank you for raising this point. We chose the 90-day and 1-year intervals based on specific literature and statistical considerations relevant to our study.

Literature Basis: The 90-day and 1-year intervals are commonly used in similar studies for evaluating outcomes in heart failure patients due to their clinical significance in assessing short-term and medium-term prognosis. For example, studies like [1, 2] have utilized these intervals to measure and compare outcomes effectively.

Statistical Justification: These intervals provide a balance between capturing significant outcomes and ensuring adequate sample sizes for robust statistical analysis. Shorter intervals may not capture long-term trends, while longer intervals may dilute the effects observed within critical periods.

We will include references and a more detailed justification in the revised manuscript to support the choice of these intervals.

**Comment 2**:

what is the p-values for the studied variables between the 2 classes.

Response:

The p-values for the studied variables between the two classes are presented in Tables 3, 4, and 5 of the Supplement. These tables provide a detailed comparison of the p-values for each variable across the two classes.

**Comment 3**:

what is tbeused CI in the Kaplan-meier plot?

Response:

Thank you for your question. The confidence intervals (CI) used in the Kaplan-Meier plot are typically calculated using the Greenwood's formula. This formula estimates the variance of the Kaplan-Meier estimator and provides the bounds for the confidence intervals.

In the revised manuscript, we will clarify the method used for calculating the confidence intervals in the Kaplan-Meier plot to ensure transparency and accuracy in our reporting.

**Comment 4**:

How English vesrus non-Ebglish matters in the prediction? justigicatio, please.

Response:

Thank you for your question. The distinction between English and non-English speakers can significantly impact prediction models due to several factors:

Communication Barriers: Non-English speakers may face difficulties in understanding medical instructions, which can affect their adherence to treatment plans and overall health management. This can lead to differences in health outcomes compared to English speakers.

Data Completeness and Accuracy: Language barriers might affect the accuracy and completeness of medical records and patient-reported outcomes. For instance, language discrepancies can lead to misinterpretation or incomplete documentation of symptoms and medical history.

Access to Care: Language can influence access to healthcare services, including preventive care and follow-up appointments. Non-English speakers might have less access to resources or face delays in receiving appropriate care, impacting their health outcomes.

Cultural Differences: Language is often linked to cultural practices and beliefs that can affect health behaviors and outcomes. Incorporating language as a factor allows for a more nuanced understanding of these influences in prediction models.

We will include a detailed justification for the inclusion of language as a factor in the prediction models in the revised manuscript, supported by relevant literature and evidence on its impact on health outcomes.

[1] Khan MS, Sreenivasan J, Lateef N, et al. Trends in 30- and 90-Day Readmission Rates for Heart Failure. Circ Heart Fail. 2021;14(4):e008335. doi:10.1161/CIRCHEARTFAILURE.121.008335

[2] Crespo-Leiro MG, Anker SD, Maggioni AP, et al. European Society of Cardiology Heart Failure Long-Term Registry (ESC-HF-LT): 1-year follow-up outcomes and differences across regions [published correction appears in Eur J Heart Fail. 2017 Mar;19(3):438. doi: 10.1002/ejhf.772]. Eur J Heart Fail. 2016;18(6):613-625. doi:10.1002/ejhf.566
