# Supplementary material for: Explainable mortality prediction models incorporating social health determinants and physical frailty for heart failure patients
Source: PLoS One. 2025 Sep 3;20(9):e0327979. doi: 10.1371/journal.pone.0327979 (PMC12407480; doi:10.1371/journal.pone.0327979)
Supplement: Attachment — Submitted filename: response_to_reviewers_auresp_2.docx [file pone.0327979.s003.docx]

**Responses to Reviewers’ Comments**

We would like to express our sincere gratitude to the editor and reviewers for their huge effort in giving excellent suggestions that would significantly improve the quality and readability of this manuscript for revising. We have studied all the comments carefully and have made corrections which we hope meet with approval.

**Responses to Reviewer #3**

**Comment 1**:

The paper use XGBoost model to predict patient mortality rates due to heart failure based on various features, such as social determinants and physical frailty. The XGBoost model outperform baseline models like GWTG-HF, SOFA, random forest and logistic regression in terms of various metrics and help identify risk factors of heart failure. The paper generally looks good. The writing needs to be polished and significantly improved.

Response: We sincerely thank the reviewer for the positive evaluation of our study and for acknowledging the contribution of our XGBoost-based model in improving heart failure mortality prediction and risk factor identification. We fully agree with the reviewer’s suggestion regarding the need to polish the manuscript. Accordingly, we have thoroughly revised the entire text for clarity, grammar, and readability. The manuscript has undergone comprehensive language editing to ensure it meets the standards of academic writing. We believe these changes have significantly improved the overall quality and presentation of our work.

**Responses to Reviewer #5**

**Comment 1**:

the manuscript has a long and monotonic introduction that has mixed up the introduction with the discussion. this will indeed reduce readability and confuse general reader.

Response: We thank the reviewer for this valuable and constructive comment. We acknowledge that the original introduction was overly long and included elements more appropriate for the discussion section, which may have affected clarity and readability. In response, we have carefully revised the introduction to make it more concise, focused, and engaging. Specifically, we have restructured the content to clearly distinguish the background, research gap, and objectives of the study, and we have removed discussion-like interpretations that were prematurely introduced. We believe the revised introduction now better aligns with academic conventions and improves the reader’s experience.
